# Intra-hospital differences in antibiotic use correlate with antimicrobial resistance rate in *Escherichia coli* and *Klebsiella pneumoniae*: a retrospective observational study

**DOI:** 10.1186/s13756-018-0387-0

**Published:** 2018-07-28

**Authors:** Alexia Cusini, David Herren, Lukas Bütikofer, Catherine Plüss-Suard, Andreas Kronenberg, Jonas Marschall

**Affiliations:** 1 Department of Infectious Diseases and Hospital Epidemiology, Bern University Hospital and University of Bern, Bern, Switzerland; 2 Institute for Infectious Diseases, University of Bern, Bern, Switzerland; 3 CTU Bern, and Institute of Social and Preventive Medicine (ISPM), University of Bern, Bern, Switzerland; 4 Service of Hospital Preventive Medicine, Lausanne University Hospital, Lausanne, Switzerland; 5 Infectious Diseases Unit, Cantonal Hospital, 7000 Chur, Switzerland

**Keywords:** *E. coli*, *K. pneumoniae*, Antibiotic resistance, Antibiotic use, Correlation

## Abstract

**Background:**

Monitoring antimicrobial use and resistance in hospitals are important tools of antimicrobial stewardship programs. We aimed to determine the association between the use of frequently prescribed antibiotics and the corresponding resistance rates in *Escherichia coli* and *Klebsiella pneumoniae* among the clinical departments of a tertiary care hospital.

**Methods:**

We performed a retrospective observational study to analyse the use of nine frequently prescribed antibiotics and the corresponding antimicrobial resistance rates in hospital acquired *E. coli* and *K. pneumoniae* isolates from 18 departments of our institution over 9 years (2008–2016). The main cross-sectional analysis assessed the hypothetical influence of antibiotic consumption on resistance by mixed logistic regression models.

**Results:**

We found an association between antibiotic use and resistance rates in *E. coli* for amoxicillin-clavulanic acid (OR per each step of 5 defined daily dose/100 bed-days 1.07, 95% CI 1.02–1.12; *p* = 0.004), piperacillin-tazobactam (OR 2.11, 95% CI 1.45–3.07; *p* < 0.001), quinolones (OR 1.52, 95% CI 1.25–1.86; *p* < 0.001) and trimethoprim-sulfamethoxazole (OR 1.59, 95% CI 1.19–2.13; *p* = 0.002). Additionally, we found a significant association when all nine antibiotics were combined in one analysis. The association between consumption and resistance rates was stronger for nosocomial than for community strains. In *K. pneumoniae,* we found an association for amoxicillin-clavulanic acid (OR 1.07, 95% CI 1.01–1.14; *p* = 0.025) and for trimethoprim-sulfamethoxazole (OR 2.02, 95% CI 1.44–2.84; *p* < 0.001). The combined analysis did not show an association between consumption and resistance (OR 1.06, 95% CI 0.99–1.14; *p* = 0.07).

**Conclusions:**

We documented an association between antibiotic use and resistance rate for amoxicillin-clavulanic acid, piperacillin-tazobactam, quinolones and trimethoprim-sulfamethoxazole in *E. coli* and for amoxicillin-clavulanic acid and trimethoprim-sulfamethoxazole in *K. pneumoniae* across different hospital departments. Our data will support stewardship interventions to optimize antibiotic prescribing at a department level.

**Electronic supplementary material:**

The online version of this article (10.1186/s13756-018-0387-0) contains supplementary material, which is available to authorized users.

## Background

Antibiotic resistance in gram-negative bacteria has increased worldwide [1]. Infections with resistant gram-negative bacteria are a particularly serious threat to public health because they are difficult to treat and are associated with high morbidity and mortality, and great healthcare costs [2].

*Escherichia coli* (*E. coli*) and *Klebsiella pneumoniae* (*K. pneumoniae*) are among the most important gram-negative pathogens in the hospital setting, accounting for around one fifth of all pathogens causing healthcare-associated infections in the USA [3]. These two pathogens are frequent causes of catheter-associated urinary tract infections (CAUTI), surgical site infections (SSI) and ventilator-associated pneumonia (VAP) [3]. Risk factors for the emergence of infections with resistant gram-negative bacteria include existing comorbidities, presence of medical devices, previous invasive procedures and admission from a long-term care facility [4]. Despite the widespread belief that the most important driver in the selection of bacterial resistance is the use of antimicrobials, the relationship between antibiotic use and resistance rates across individual departments within a single hospital is not well studied. Willemsen et al. comprehensively investigated this relationship in a teaching hospital in the Netherlands and showed a correlation between ward-specific use and resistance rates for ciprofloxacin and amoxicillin-clavulanic acid in *E. coli* [5]. Compared to Willemsen et al. we have investigated resistance data for *K. pneumoniae* in addition to *E. coli*, included more antibiotics and used the annual antibiotic consumption per 100 bed-days per department over 9 years (2008–2016). Our objective was to identify antibiotics associated with a higher risk of resistance development in order to optimise the antibiotic prescribing practice particularly in those departments in our hospital with the highest antibiotic consumption.

## Methods

### Study aim

We aimed to determine the association between the use of frequently prescribed antibiotics and the corresponding resistance rates in *E. coli* and *K. pneumoniae* among the clinical departments of a tertiary care hospital.

### Study design

We performed a retrospective observational study of antibiotic use and antibiotic resistance rates of *E. coli* and *K. pneumoniae* across 18 departments at the Bern University Hospital in Bern, Switzerland, between January 1st 2008 and December 31st 2016.

### Hospital setting

Bern University Hospital is a 950-bed tertiary care teaching hospital that covers all medical specialties and includes a 30-bed mixed intensive care unit (ICU). There are around 40′000 admissions annually, resulting in 290′000 patient-days. The following 18 departments were included in the study: abdominal surgery and medicine, cardiology, cardiovascular surgery, critical care, dermatology, general internal medicine, gynecology and obstetrics, nephrology, neurology, neurosurgery, oncology and hematology, ophthalmology, orthopedics, otorhinolaryngology, plastic and hand surgery, rheumatology and clinical immunology, thoracic surgery and pulmonology, and urology.

### Data collection

Data was extracted from Anresis [6], the national surveillance program that collects data on antibiotic resistance and antibiotic consumption in all Swiss hospitals, including Bern University Hospital.

Antimicrobial susceptibility testing (AST) for the following frequently prescribed antibiotics active against wild type *E. coli* and *K. pneumoniae* were included: amoxicillin (only active against *E. coli*), amoxicillin-clavulanic acid, ceftriaxone, cefepime, piperacillin-tazobactam, meropenem, ciprofloxacin, norfloxacin, trimethoprim-sulfamethoxazole and gentamicin. Antibiotics mainly prescribed for perioperative prophylaxis (e.g. cefuroxime) were not included. All *E. coli* and *K. pneumoniae* isolates originated from routine clinical diagnostics. AST for isolates from urine was routinely performed only for amoxicillin, amoxicillin-clavulanic acid, quinolones and trimethoprim-sulfamethoxazole; all other isolates were tested for all listed antibiotics. Therefore, the number of tested samples varies for each of the antibiotics. AST was performed at the Institute for Infectious Diseases of the University of Bern and was based on breakpoints for inhibitory zone diameters (disk diffusion method) according to the guidelines of the Clinical and Laboratory Standards Institute (CLSI). Over the study period the approved version of the 9th, 10th and 11th CLSI edition were adopted by the laboratory. Intermediate susceptibility was recorded as resistance. Quinolone resistance was defined as resistance to at least one of the substances in this group (e.g. norfloxacin and/or ciprofloxacin). We included only the last sample per patient per calendar year. In the main analysis, we included only hospital-acquired samples, i.e. samples that had been isolated more than 2 days after hospital admission. In subsequent sensitivity analyses for community-acquired samples, only samples isolated during the first two hospitalization days were analysed. In addition, we repeated the analysis using a stricter definition of hospital-acquired infection including only the bacterial isolates that had been detected more than 5 days after hospital admission.

The hospital pharmacy provided data on antibiotic consumption in numbers of packages delivered to each department from 2008 to 2016. We assumed that deliveries to the departments reflected consumption as all unused antibiotics were returned to the pharmacy and subtracted for our analyses. All departments used the same delivery system without significant storage and without shifting of antibiotics from one department to another. In the Anresis database the aggregated data were converted into defined daily doses (DDD) using the ATC/DDD system promoted by the World Health Organization [7]. The DDD is the assumed average maintenance dose per day for an antimicrobial used for its main indication in adults.

We obtained annual bed-days per department from the finance and controlling department of Bern University Hospital. In their calculation, the day of admission and the day of discharge were counted together as one bed-day. Annual Antibiotic use in DDD/100 bed-days was calculated for each of the 18 departments.

### Statistical analyses

Antibiotic use across the departments was summarized by median, interquartile range (IQR, lower quartile-upper quartile) and total range (minimum-maximum) of the time-averaged data. The change in antibiotic use over time within each department was analysed with a linear regression model with year, department and the interaction of year and department as covariates. The marginal mean per department and the change per year and department are presented as DDD/100 bed-days with 95% confidence intervals (CI).

We used mixed logistic regression to model antibiotic resistance in dependence of consumption. The main analysis was cross-sectional (i.e. consumption and resistance were assessed at the same time) and was restricted to nosocomial strains. We fitted antibiotic-specific models that only considered resistance towards and use of a specific antibiotic and a combined model that considered data from all antibiotics and treated the antibiotic as random effect. The antibiotic-specific model included a fixed effect for antibiotic use and random intercepts for department and year (nested within department). The combined model included a fixed effect for antibiotic use and random intercepts for antibiotic, department (nested within antibiotic), and year (nested within antibiotic and department). Overall resistance proportions and corresponding 95% CI were obtained by marginal predictions. The association of consumption and resistance is expressed as an odds ratio (OR) per each step of 5 DDD/100 bed-days (i.e. referring to the incremental change in the odds for resistance for an increase in consumption by 5 DDD/100 bed-days) with corresponding 95% CI.

We performed four sensitivity analyses. First, resistance was regressed on the antibiotic use of the preceding year to investigate a potential temporal relation. Second, we repeated the analysis for community-acquired bacterial strains to check for a potential spillover of the (hospital) consumption effect. The strength of the association was compared with that found for nosocomial strains by fitting models using both nosocomial and community data with an additional random intercept for the mode of acquisition. We compared models with and without the interaction of antibiotic use and the site of acquisition using likelihood ratio tests; the *p*-value is reported as *p*-value for interaction. Third, we performed the analysis using a stricter definition of hospital-acquired infection including only the strains that were obtained more than 5 days after hospital admission in the analysis. Fourth, we performed separate analyses for isolates from urine, blood and other sites of origin. We calculated a *p*-value for interaction from likelihood ratio tests of models with and without an interaction for consumption and the origin of the sample. All analyses were done with Stata version 15 (StataCorp. 2017. Stata Statistical Software: Release 15. College Station, TX.)

## Results

### Antibiotic consumption in 18 individual hospital departments

The average use of the antibiotics selected for this study was 48.5 DDD/100 bed-days, corresponding to 62% of the total antibiotic use in the study departments between 2008 and 2016.

**Fig. 1:**
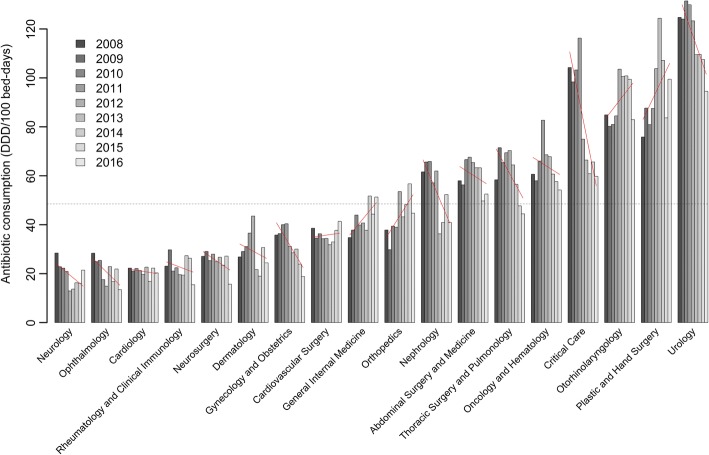
Annual use of the study antibiotics in DDD/100 bed days in 18 departments for the period between 2008 and 2016. The red lines indicate fitted values from a linear regression model, the dashed line the overall average

The annual use of these antibiotics varied widely between the 18 departments, from a mean of 19.4 (95% CI: 14.7–24.2) DDD/100 bed-days in neurology to a mean of 117 (95% CI: 112–122) DDD/100 bed-days in urology (Fig. 1 and Additional file 1: Table S1). Within the departments the antibiotic use changed unequally over the study period. It decreased significantly in critical care, gynecology and obstetrics, nephrology, thoracic surgery/pulmonology and urology departments, and increased significantly in the orthopedics, plastic and hand surgery departments. The trends in antibiotic use within each department are illustrated by fitted lines in Fig. 1 and summarised in Additional file 1: Table S1.

Overall, amoxicillin-clavulanic acid was by far the most frequently used antibiotic with a median use of 19.0 DDD/100 bed-days (IQR: 10.0–26.5), followed by cefepime with 3.9 DDD/100 bed-days (IQR 1.7–5.2) and ceftriaxone with 2.6 DDD/100 bed-days (IQR: 2.0–5.3) (Table 1).

**Table 1:** Annual antibiotic consumption (DDD/100 bed-days) summarized across 18 departments in the years 2008–2016

	Median	Interquartile range	Total range
Amoxicillin/Ampicillin	2.7	1.2–5.2	0.3–8.4
Amoxicillin-clavulanic acid	19.0	10.0–26.5	6.4–85.3
Ceftriaxone	2.6	2.0–5.3	0.5–8.7
Cefepime	3.9	1.7–5.2	0.1–30.2
Piperacillin-tazobactam	0.9	0.4–1.8	0.0–11.1
Meropenem	1.2	0.3–2.7	0.1–9.8
Quinolones	2.4	1.8–3.5	0.8–15.4
Trimethoprim-sulfamethoxazole	1.7	1.0–3.2	0.2–13.3
Gentamicin	0.2	0.1–0.5	0.0–2.4

### Antibiotic resistances in *E. coli* isolates

The different origins (urine, blood, respiratory tract, wound swabs and other) of the *E. coli* isolates, the number of AST performed for each antibiotic and the time point of sampling during the hospital stay are shown in Table 2. Sixty-five percent of the isolates originated from urine and 11% from blood. For amoxicillin/ampicillin, amoxicillin-clavulanic acid, quinolones and trimethoprim-sulfamethoxazole, 139 department-years with 2329 (trimethoprim-sulfamethoxazole) and 2330 (other antibiotics) samples, were analysed. For ceftriaxone, cefepime, piperacillin-tazobactam and gentamicin we analyzed 119 department-years with 1083 (cefepime) and 1085 (other antibiotics) samples. The resistance rate was highest for amoxicillin/ampicillin at 62% (95% CI 55–69%), followed by amoxicillin-clavulanic acid at 39% (95% CI 35–44%), and trimethoprim-sulfamethoxazole at 32% (95% CI 27–37%) (Table 3). For all tested antibiotics the resistance rates for hospital-acquired samples was higher than for community-acquired samples (Additional file 1: Table S3).

**Table 2:** Number of *E. coli* samples with antimicrobial susceptibility testing (AST) by origin across 18 departments in the years 2008–2016

Origin of the samples	Tested for	All nosocomial samples (*N* = 2330)	Samples obtained after 2 to 5 hospital days (*N* = 525)	Samples obtained after 5 hospital days (*N* = 1805)
Urine	AM-CL, Amox/AMP, Quinolones, TMP-SMX	1503 (65%)	377 (72%)	1126 (62%)
	CFP	256 (11%)	53 (10%)	203 (11%)
	CFT, Gent, MER^a^, PIP-TZ,	258 (11%)	55 (10%)	203 (11%)
Blood	AM-CL, Amox/AMP, CFP, CFT, Gentamicin, MER^a^, PIP-TZ, Quinolones, TMP-SMX	264 (11%)	32 (6%)	232 (13%)
Respiratory -tract	AM-CL, Amox/AMP, CFP, CFT, Gent, MER^a^, PIP-TZ, Quinolones, TMP-SMX	86 (4%)	29 (6%)	57 (3%)
Wound swabs	AM-CL, Amox/AMP, CFP, CFT, Gent, MER^a^, PIP-TZ, Quinolones	83 (4%)	12 (2%)	71 (4%)
	TMP-SMX	82 (4%)	12 (2%)	70 (4%)
Other	AM-CL, Amox/AMP, CFP, CFT, Gent, MER^a^, PIP-TZ, Quinolones, TMP-SMX	394 (17%)	75 (14%)	319 (18%)

*Abbreviations*: *Amox* amoxicillin, *AMP* ampicillin, *AM-CL* amoxicillin-clavulanic acid, *CFP* cefepime, *CFT* ceftriaxone, *Gent* gentamicin, *MER* meropenem, *PIP-TZ* piperacillin-tazobactam, *TMP-SMX* trimethoprim-sulfamethoxazole

^a^Not included in the models for resistance because all samples were susceptible to Meropenem

**Table 3:** Antibiotic resistance and association with antibiotic use for *E. coli*

	Resistance		Association with antibiotic use	
	No. of tests/strata	Proportion resistant (95% CI)	Odds ratio (95% CI)	*p*-value
Amoxicillin/Ampicillin	2330 / 139	62 (55–69)	1.08 (0.84–1.40)	0.53
Amoxicillin-clavulanic acid	2330 / 139	39 (35–44)	1.07 (1.02–1.12)	0.004
Ceftriaxone	1085 / 119	18 (13–24)	1.10 (0.68–1.76)	0.71
Cefepime	1083 / 119	8 (6–12)	1.06 (0.90–1.25)	0.49
Piperacillin-tazobactam	1085 / 119	17 (13–21)	2.11 (1.45–3.07)	< 0.001
Quinolones	2330 / 139	20 (17–23)	1.52 (1.25–1.86)	< 0.001
Trimethoprim-sulfamethoxazole	2329 / 139	32 (27–37)	1.59 (1.19–2.13)	0.002
Gentamicin	1085 / 119	18 (14–22)	1.30 (0.45–3.71)	0.63
Combined	13,657 / 1032	24 (15–36)	1.09 (1.04–1.14)	< 0.001

Results from logistic mixed models for antibiotic resistance in relation to consumption. Odds ratios represent the change in the odds for resistance per increase in antibiotic use by 5 DDD/100 bed-days. Strata refer to year/department combinations

*CI* confidence interval

### Association of antibiotic consumption and resistance in *E. coli* isolates

**Fig. 2:**
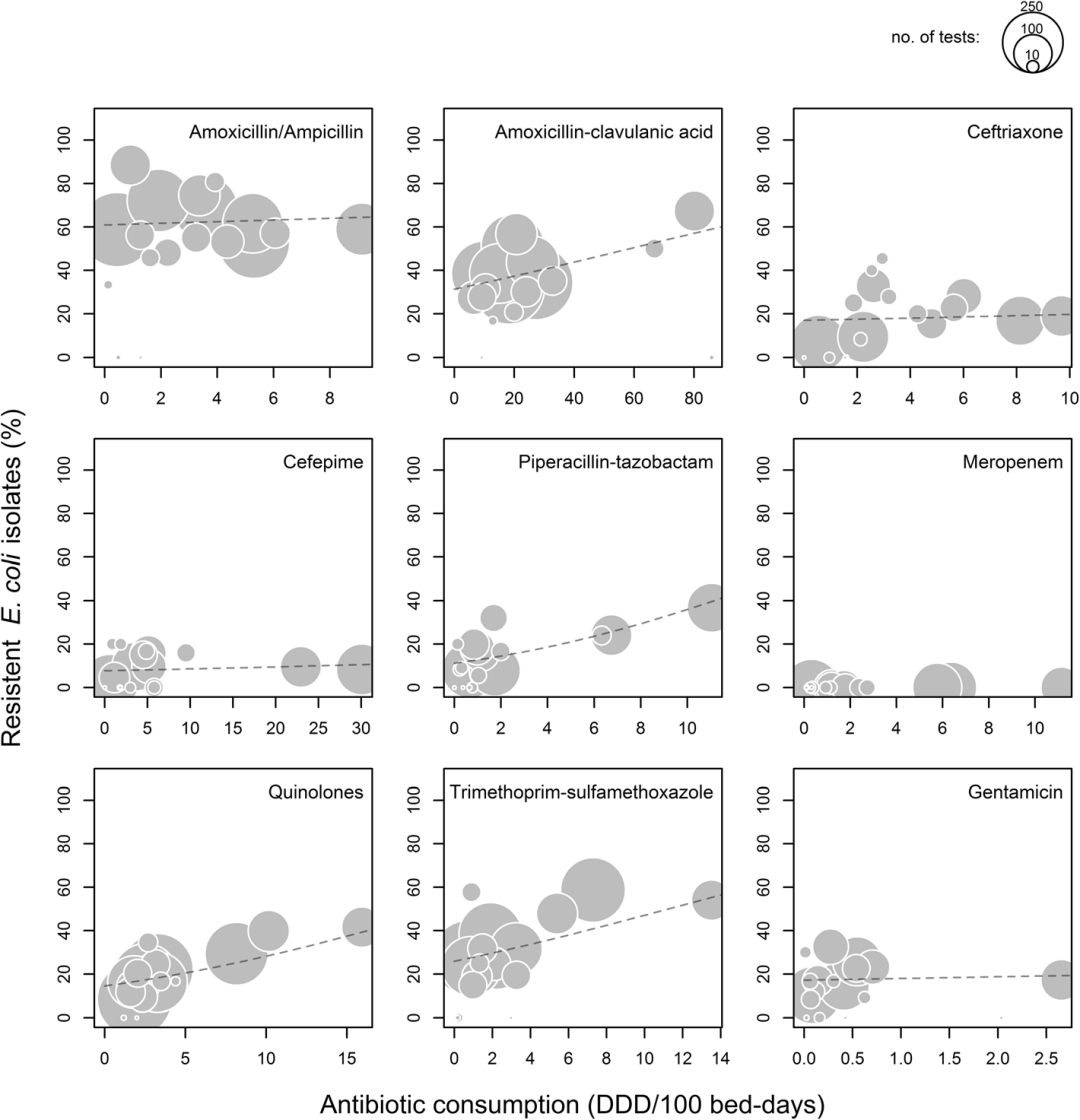
Comparison of the proportions of resistant nosocomial *E. coli* and antibiotic use. Each point indicates the time-averaged values for an individual department for the period between 2008 and 2016. The size corresponds to the number of bacteria tested for resistance, i.e. reflects the precision of the resistance estimate. The dashed lines indicate the fitted value from the logistic mixed models

We found a significant positive association between use and resistance rates in *E. coli* for amoxicillin-clavulanic acid (OR per each step of 5 DDD/100 bed-days 1.07, 95% CI 1.02–1.12; *p* = 0.004), piperacillin-tazobactam (OR 2.11, 95% CI 1.45–3.07; *p* < 0.001), quinolones (OR 1.52, 95% CI 1.25–1.86; *p* < 0.001) and trimethoprim-sulfamethoxazole (OR 1.59, 95% CI 1.19–2.13; *p* = 0.002). An association between antibiotic use and antimicrobial resistance was also found in the combined analysis (OR 1.09, 95% CI 1.04–1.14; *p* < 0.001). No significant association between use and resistance rates in *E. coli* were found for amoxicillin/ampicillin, ceftriaxone, cefepime or gentamicin. (Table 3, Fig. 2). Due to the absence of meropenem-resistant strains of *E. coli* the association between meropenem use and the resistance rate could not be studied.

The association between antimicrobial resistance and the preceding year’s antibiotic consumption was similar to the analysis for all study antibiotics (Additional file 1: Table S2). The association of antibiotic use and resistance appeared to be stronger in hospital-acquired than in community-acquired strains for most antibiotics and a significant effect of resistance on consumption in community strains was only found for trimethoprim-sulfamethoxazole However, we could hardly prove systematic differences and a significant interaction was only found for piperacillin-tazobactam (*p* = 0.034) (Additional file 1: Table S3).

Using a stricter definition for hospital-acquired strains — by only considering samples obtained more than 5 days after hospital admission — did not have significant influence on the results and all main findings were identical (Additional file 1: Table S4). Analysis of subgroups according to sample origin confirmed an association of antibiotic use and resistance for amoxicillin/clavulanic acid, quinolones, trimethoprim-sulfamethoxazole and for the combined analysis in urine. An association for piperacillin-tazobactam was only found in the samples other than urine and blood (Additional file 1: Table S5). However, we did not find much evidence for systematic differences and a significant *p*-value for interaction was only observed for gentamicin.

### Antibiotic resistance in *K. pneumoniae* isolates

For amoxicillin-clavulanic acid, quinolones and trimethoprim-sulfamethoxazole we included 115 department-years with 648 samples; for cefepime, ceftriaxone, gentamicin and piperacillin-tazobactam 92 department-years with 369 samples. Fifty one percent of the *K. pneumoniae* samples were isolated from urine, 14% from blood and 11% from the respiratory tract (Table 4). The resistance rate was highest for piperacillin-tazobactam at 21% (95% CI 17–25%), followed by amoxicillin-clavulanic acid at 17% (95% CI 14–21%) and trimethoprim-sulfamethoxazole at 15% (95% CI 12–19%) (Table 5).

**Table 4:** Number of *K. pneumoniae* samples with antimicrobial susceptibility testing (AST) by origin across 18 departments in the years 2008–2016

Origin of the samples	Tested for	All nosocomial samples (*N* = 648)	Samples obtained after 2 to 5 hospital days (*N* = 124)	Samples obtained after 5 hospital days (*N* = 524)
Urine	AM-CL, Amox/AMP^b^, Quinolones, TMP-SMX	329 (51%)	72 (58%)	257 (49%)
	CFP, CFT, Gent, MER^a^, PIP-TZ	51 (8%)	12 (10%)	39 (7%)
Blood	AM-CL, Amox/AMP^b^, CFP, CFT, Gent, MER^a^, PIP-TZ, Quinolones, TMP-SMX	89 (14%)	9 (7%)	80 (15%)
Respiratory samples	AM-CL, Amox/AMP^b^, CFP, CFT, Gent, MER^a^, PIP-TZ, Quinolones, TMP-SMX	69 (11%)	16 (13%)	53 (10%)
Wound swabs	AM-CL, Amox/AMP^b^, CFP, CFT, Gent, MER^a^, PIP-TZ, Quinolones, TMP-SMX	22 (3%)	3 (2%)	19 (4%)
Other	AM-CL, Amox/AMP^b^, CFP, CFT, Gent, MER^a^, PIP-TZ, Quinolones, TMP-SMX	139 (21%)	24 (19%)	115 (22%)
	PIP-TZ	138 (21%)	24 (19%)	114 (22%)

*Abbreviations*: *Amox* amoxicillin, *AMP* ampicillin, *AM-CL* amoxicillin-clavulanic acid, *CFP* cefepime, *CFT* ceftriaxone, *Gent* gentamicin, *MER* meropenem, *PIP-TZ* piperacillin-tazobactam, *TMP-SMX* trimethoprim-sulfamethoxazole

^a^Not included in the models for resistance because only two samples were resistant to Meropenem

^b^Not included in the models for resistance because all samples were resistant to Amoxicillin/Ampicillin

**Table 5:** Antibiotic resistance and association with antibiotic use for *K. pneumoniae*

	Resistance		Association with antibiotic use	
	No. of tests/strata	Proportion resistant (95% CI)	Odds ratio (95% CI)	*p*-value
Amoxicillin-clavulanic acid	648 / 115	17 (14–21)	1.07 (1.01–1.14)	0.025
Ceftriaxone	369 / 92	10 (5–18)	1.12 (0.52–2.45)	0.77
Cefepime	369 / 92	3 (1–9)	0.86 (0.59–1.25)	0.43
Piperacillin-tazobactam	369 / 92	21 (17–25)	0.84 (0.61–1.18)	0.32
Quinolones	648 / 115	6 (3–9)	1.37 (0.94–2.01)	0.10
Trimethoprim-sulfamethoxazole	648 / 115	15 (12–19)	2.02 (1.44–2.84)	< 0.001
Gentamicin	369 / 92	8 (4–16)	1.82 (0.13–25.42)	0.66
Combined	3420 / 713	12 (8–16)	1.06 (0.99–1.14)	0.07

Results from logistic mixed effects models. The odds ratios represent the relative change in the odds for resistance for an increase in antibiotic use by 5 DDD/100 bed-days. Strata refers to year/department combinations

*CI* confidence interval

### Association of antibiotic consumption and resistance in *K. pneumoniae* isolates

**Fig. 3:**
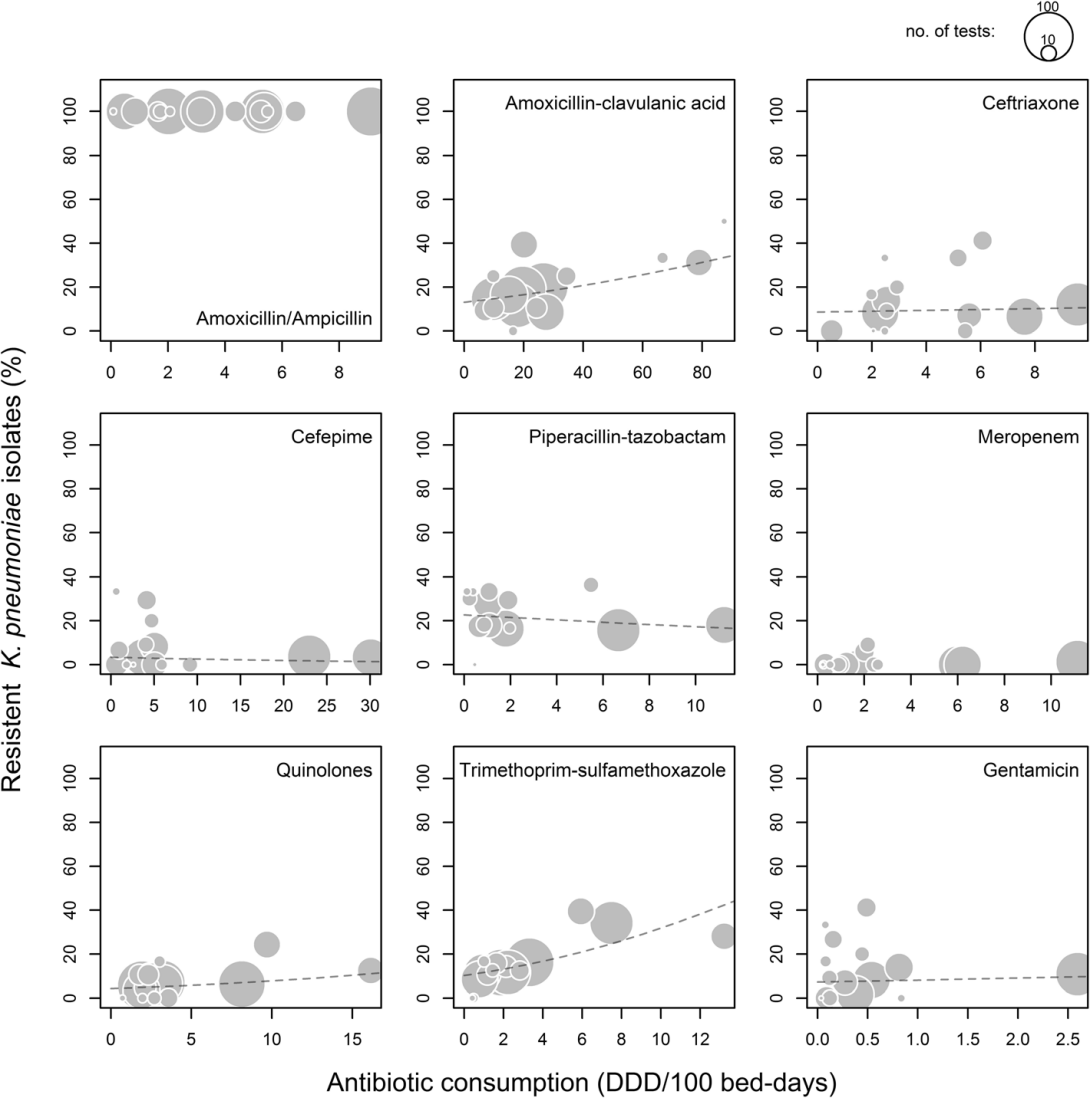
Comparison of the proportions of resistant nosocomial K. pneumoniae and antibiotic use. Each point indicates the time-averaged values for an individual department for the period between 2008 and 2016. The size corresponds to the number of bacteria tested for resistance, i.e. reflects the precision of the resistance estimate. The dashed lines indicate the fitted value from the logistic mixed models

There was a significant association between use and resistance rates for amoxicillin-clavulanic acid (OR per 5 DDD/100 patient-days 1.07, 95% CI 1.01–1.14; *p* = 0.025) and trimethoprim-sulfamethoxazole (OR 2.02, 95% CI 1.44–2.84; *p* < 0.001) No association between use and resistance rates were found for ceftriaxone, cefepime, piperacillin-tazobactam, quinolones or gentamicin. The combined analysis did not show an overall association between consumption and resistance (Table 5, Fig. 3).

We did not analyse meropenem and amoxicillin/ampicillin because only two meropenem-resistant strains were observed and *K. pneumoniae* has an intrinsic resistance to amoxicillin/ampicillin.

Using last year’s antibiotic use did not show any major differences to the main analysis (Additional file 1: Table S6). In community strains, we observed an association between antibiotic use and resistance for trimethoprim-sulfamethoxazole (OR 1.62, 95% CI 1.08–2.42; *p* = 0.021) and for quinolones (OR 1.42, 95% CI 1.04–1.96; *p* = 0.029). Evidence for an interaction was not found (Additional file 1: Table S7).

Using a stricter definition for hospital-acquired strains largely confirmed the main analysis but the ORs tended to be increased and the combined analysis was now significant (OR 1.09, 95% CI 1.02–1.18; *p* = 0.012) (Additional file 1: Table S8). In the sample origin subgroups, we only found significant associations for trimethoprim-sulfamethoxazole use and resistance in urine and blood samples. We did not observe much evidence for an influence of sample origin on the relationship of use and resistance (Additional file 1: Table S9).

## Discussion

Our analysis showed an association between use and resistance for amoxicillin-clavulanic acid, piperacillin-tazobactam, quinolones and trimethoprim-sulfamethoxazole in *E. coli* and for amoxicillin-clavulanic acid and trimethoprim-sulfamethoxazole in *K. pneumoniae* across different hospital departments.

It is intuitive that antibiotic resistance emerges predominantly in environments with high antibiotic consumption. This has been shown for individual countries [8] and individual hospitals [9–11]. It is however conceivable that even antibiotic prescription within individual departments of the same hospital may affect bacterial resistance in the patients sharing the same environment, as bacteria can be transmitted from patient to patient and antibiotic resistance can spread between bacteria. In this regard, the results of our study confirmed the main findings reported by Willemsen et al. demonstrating that a higher use of amoxicillin-clavulanic acid and quinolones was associated with higher antimicrobial resistance in *E. coli*.

Interestingly, we only found associations between antibiotic use and resistance for some antibiotics and different associations between *E. coli* and *K. pneumoniae*.

A plausible explanation is the considerable difference in the quantitative use of the antibiotics studied across our hospital’s departments. Considering the substantial use of amoxicillin-clavulanic acid at our institution, it is not surprising that its use was associated with resistance in both microorganisms analysed. A wide quantitative range of antibiotic use across different departments may also facilitate the detection of an association, as observed for piperacillin/tazobactam, where the median consumption was low, but the range was very wide.

However, the resistance barrier of the individual antibiotics may also be relevant for the emergence of resistance. For cefepime, which is recommended in our internal antibiotic guidelines [12] as first-line antibiotic for several nosocomial infections (e.g. pneumonia and fever in neutropenia), we did not find an association between use and resistance, even though it was the second most frequently used antibiotic overall. Cefepime is a robust fourth generation cephalosporin, which is relatively stable against β-lactamases and maintains activity even against difficult organisms. In contrast, *Enterobacteriaceae* may acquire resistance to quinolones and trimethoprim-sulfamethoxazole more readily and we indeed observed significant associations between their use and resistance in *E. coli.*

Results for *K. pneumoniae* were less clear than for *E. coli*, mainly because the sample size was much smaller. In particular, for ceftriaxone, cefepime, piperacillin-tazobactam and gentamicin the number of susceptibility tests performed for *K. pneumoniae* (*n* = 369) was substantially lower than for *E. coli* (*n* = 1085).

Our first sensitivity analysis examined the potential temporal relationship between the consumption of antibiotics and the development of resistance a year later. However, the last year’s use did not have a stronger influence than the current use. Possible explanations are the relatively stable antibiotic use over time in our setting or a different timing of the emergence of resistance. Even a reverse causation is possible — the reduction in use due to the emergence of resistance.

The antibiotic use varied widely between the different departments and within the individual departments; there were significant variations in antibiotic use over time. Notably, the antibiotic use was high in predominantly surgical departments like urology, plastic and hand surgery, and otorhinolaryngology. Even though cefuroxime, which is recommended for perioperative prophylaxis, was not included in the analysis the high antibiotic use of these departments is probably due to the frequent use of amoxicillin-clavulanic acid and quinolones (urology) for perioperative prophylaxis that in some cases is given longer than suggested by international guidelines. During the study period, there were no specific antimicrobial stewardship interventions in any hospital department. Improving perioperative prophylaxis in the mentioned surgical departments will be a priority in the future.

To address the specificity of the association we compared nosocomial and community strains, given that the latter was not exposed to antibiotic use in the hospital. As expected, the association between antibiotic consumption and resistance tended to be systematically stronger for nosocomial strains than for community strains in *E. coli* even though we did not always find significant interactions. This result is consistent with another Swiss study showing that strains isolated ≥48 h after admission were less susceptible than those stemming from the community [13]. The same pattern was not found for *K. pneumoniae*, mainly because we did not observe many clear associations in the first place. Trimethoprim-sulfamethoxazole, for which the association persisted in community-acquired *K. pneumoniae* strains, is also frequently prescribed in the outpatient setting [14]. Furthermore, we could not distinguish patients with previous contacts with the healthcare system and some community-acquired infections could actually be healthcare-associated.

The accuracy of distinguishing community- from hospital-acquired pathogens plays an important role in our analyses. We chose a 2-day cut-off according to the Centers for Disease Control and Prevention (CDC) definition [15]. A stricter definition for hospital-acquired samples (i.e. a cut-off of five instead of 2 days after hospital admission) did not change any of the main findings, as the vast majority of samples (79% for *E. coli* and 81% for *K. pneumoniae*) was obtained after 5 days of hospital admission.

In recent years, several studies have analyzed the relationship between antibiotic use and development of resistance in *Enterobacteriaceae*. Consistent with our findings, several studies have described an association between in-hospital antibiotic use and resistance to quinolones in *E. coli* [16–18]*.* This association may be particularly relevant for Switzerland with its comparatively higher quinolone consumption than other European countries [19].

Two other studies have reported associations in *E. coli* between amoxicillin-clavulanic acid [5, 20] and piperacillin-tazobactam [21, 22]. The main discrepancy between our results and those of other studies is the absence of an association between the use of ceftriaxone and the emergence of third-generation cephalosporin resistance in our study [20, 23, 24]. This may be due to a relatively low median use of ceftriaxone of 2.6 DDD/100 patient days compared to other studies.

Our study has several limitations. First, the number of AST available differed considerably among the antibiotics and the sample size was low for some. The majority of samples (65% of *E. coli*, and 51% of *K. pneumoniae*) originated from urine and a complete AST of these samples is only performed if routine testing shows several resistances. Therefore, the resistance data for cefepime, ceftriaxone, piperacillin-tazobactam and gentamicin was only available in one sixth of the urine samples. The low sample size reduces the power of the analysis, which was particularly problematic for *K. pneumoniae* where it severely hampered the conclusions*.* Moreover testing some antibiotics only in higher resistant isolates may lead to an overestimation of resistance in urinary samples against these antibiotics, due to sampling bias. Second, this was a single-centre study, therefore our results may not be generalizable to other contexts with potential differences in case-mix and epidemiologic characteristics. Third, we could not take into account individual risk factors for infections with resistant microorganisms (such as underlying diseases or previous antimicrobial therapy), because of the anonymization of the samples. Fourth, we did not analyse the association between the use of one particular antibiotic and the resistance to a second antibiotic. Fifth, the antibiotic consumption expressed in DDD per 100 bed-days does not reflect the adequacy of treatment in terms of proper dosage and duration. Sixth, we calculated antibiotic use based on deliveries to the hospital departments rather than administration to the patients. The departments of our hospital are advised to order only those antibiotics that are needed for daily use. Antibiotics that are delivered to the departments but are for some reason not administrated to the patients must be returned to the hospital pharmacy. We subtracted the returned antibiotics from the deliveries and are confident that our data reflect the real antibiotic administration to the patients.

However, to the best of our knowledge, our study is the first comprehensive analysis of the use of the most important antibiotics for the treatment of gram-negative bacteria and corresponding antimicrobial resistance across multiple departments within one institution. We included all clinical *E. coli* and *K. pneumoniae* samples isolated across 18 departments at our institution over the study period of 9 years and used the well-established, standardized ATC/DDD system. The study therefore has important relevance for clinical practice.

## Conclusions

We have demonstrated that antibiotic use in different hospital departments is associated with resistance rates in *E. coli* and in *K. pneumoniae* for some but not all antibiotics and antibiotics differ in the strength of the association. Despite a high consumption of cefepime we could not demonstrate an association between its use and resistance rates. We will therefore continue to promote the use of cefepime over piperacillin/tazobactam as an empiric broad-spectrum antibiotic in our guidelines.

Keeping resistance in *Enterobacteriaceae* in check requires a comprehensive approach including infection control strategies and an antibiotic stewardship program. Our data support targeted antibiotic stewardship interventions, such as department-specific or antimicrobial agent-specific interventions, to improve antimicrobial prescribing and reduce the development of antimicrobial resistance.

## Additional file

Additional file 1: **Table S1.** Mean antibiotic use and change per year in 18 departments in years 2008-2016. **Table S2.** Antibiotic resistance and association with last year's antibiotic use for *E. coli*. **Table S3.** Antibiotic resistance and association with antibiotic use for nosocomial and community *E. coli*. **Table S4.** Antibiotic resistance and association with antibiotic use for *E. coli* with an alternative definition of nosocomial. **Table S5.** Antibiotic resistance in *E. coli* and association with antibiotic use according to the origin of the sample. **Table S6.** Antibiotic resistance and association with last year's antibiotic use for *K. pneumoniae*. **Table S7.** Antibiotic resistance and association with antibiotic use for nosocomial and community *K. pneumoniae.* **Table S8.** Antibiotic resistance and association with antibiotic use for *K. pneumoniae* with an alternative definition of nosocomial. **Table S9.** Antibiotic resistance in *K. pneumoniae* and association with antibiotic use according to the origin of the sample. (DOCX 77 kb)

## Abbreviations

AM-CL: Amoxicillin-clavulanic acid
Amox: Amoxicillin
AMP: Ampicillin
AST: Antimicrobial susceptibility testing
CAUTI: Catheter-associated urinary tract infections
CDC: Centers for Disease Control and Prevention
CFP: Cefepime
CFT: Ceftriaxone
CI: Confidence interval
CLSI: Clinical and Laboratory Standards Institute
DDD: Defined daily doses
*E. coli*: *Escherichia coli*
ESBL: Extended-spectrum beta lactamase
Gent: Gentamicin
ICU: Intensive care unit
IQR: Interquartile range
*K. pneumoniae*: *Klebsiella pneumoniae*
MER: Meropenem
OR: Odds ratio
PIP-TZ: Piperacillin-tazobactam
SSI: Surgical site infections
TMP-SMX: Trimethoprim-sulfamethoxazole
VAP: Ventilator-associated pneumonia
WHO: World Health Organisation
