# Supplementary material for: Intra-hospital differences in antibiotic use correlate with antimicrobial resistance rate in Escherichia coli and Klebsiella pneumoniae: a retrospective observational study
Source: Antimicrob Resist Infect Control. 2018 Jul 28;7:89. doi: 10.1186/s13756-018-0387-0 (PMC6064170; doi:10.1186/s13756-018-0387-0)
Supplement: Supplementary file 1 — Table S1. Mean antibiotic use and change per year in 18 departments in years 2008-2016. Table S2. Antibiotic resistance and association with last year's antibiotic use for E. coli. Table S3. Antibiotic resistance and association with antibiotic use for nosocomial and community E. coli. Table S4. Antibiotic resistance and association with antibiotic use for E. coli with an alternative definition of nosocomial. Table S5. Antibiotic resistance in E. coli and association with antibiotic use according to the origin of the sample. Table S6. Antibiotic resistance and association with last year's antibiotic use for K. pneumoniae. Table S7. Antibiotic resistance and association with antibiotic use for nosocomial and community K. pneumoniae. Table S8. Antibiotic resistance and association with antibiotic use for K. pneumoniae with an alternative definition of nosocomial. Table S9. Antibiotic resistance in K. pneumoniae and association with antibiotic use according to the origin of the sample. (DOCX 77 kb) [file 13756_2018_387_MOESM1_ESM.docx]

**Additional file 1**

| **Table S1.** Mean antibiotic use and change per year in 18 departments in years 2008-2016. | | | |  |  |  |
| --- | --- | --- | --- | --- | --- | --- |
| Department | Mean (95% CI) | Change per year (95% CI) | P-value |  |  |  |
| Abdominal Surgery and Medicine | 60.3 (55.5 to 65.0) | -0.87 (-2.71 to 0.96) | 0.35 |  |  |  |
| Cardiology | 20.9 (16.2 to 25.7) | -0.22 (-2.06 to 1.62) | 0.81 |  |  |  |
| Cardiovascular Surgery | 35.8 (31.0 to 40.5) | 0.19 (-1.65 to 2.03) | 0.84 |  |  |  |
| Critical Care | 83.3 (78.6 to 88.1) | -6.85 (-8.68 to -5.01) | <0.001 |  |  |  |
| Dermatology | 29.2 (24.5 to 34.0) | -0.73 (-2.57 to 1.11) | 0.43 |  |  |  |
| General Internal Medicine | 42.5 (37.7 to 47.2) | 1.65 (-0.19 to 3.49) | 0.08 |  |  |  |
| Gynecology and Obstetrics | 31.7 (26.9 to 36.4) | -2.29 (-4.13 to -0.45) | 0.015 |  |  |  |
| Nephrology | 53.6 (48.9 to 58.4) | -3.22 (-5.06 to -1.38) | <0.001 |  |  |  |
| Neurology | 19.4 (14.7 to 24.2) | -1.13 (-2.97 to 0.71) | 0.23 |  |  |  |
| Neurosurgery | 25.3 (20.5 to 30.0) | -0.94 (-2.78 to 0.90) | 0.31 |  |  |  |
| Oncology and Hematology | 64.0 (59.3 to 68.8) | -0.87 (-2.71 to 0.97) | 0.35 |  |  |  |
| Ophthalmology | 20.7 (15.9 to 25.4) | -1.34 (-3.18 to 0.50) | 0.15 |  |  |  |
| Orthopedics | 43.6 (38.9 to 48.4) | 2.17 (0.33 to 4.01) | 0.021 |  |  |  |
| Otorhinolaryngology | 90.9 (86.1 to 95.6) | 1.76 (-0.08 to 3.60) | 0.06 |  |  |  |
| Plastic and Hand Surgery | 94.5 (89.7 to 99.2) | 2.86 (1.02 to 4.70) | 0.003 |  |  |  |
| Rheumatology and Clinical Immunology | 22.7 (18.0 to 27.5) | -0.52 (-2.36 to 1.32) | 0.58 |  |  |  |
| Thoracic Surgery and Pulmonology | 60.9 (56.1 to 65.6) | -2.49 (-4.33 to -0.65) | 0.008 |  |  |  |
| Urology | 117 (112 to 122) | -3.91 (-5.75 to -2.07) | <0.001 |  |  |  |
| Results from separate linear regression models for each department indicating mean antibiotic use and absolute change in DDD/100 bed-days per year. | | | |  |  |  |

| **Table S2.** Antibiotic resistance and association with last year's antibiotic use for *E. coli* | | | | | | |  |  |  |  |
| --- | --- | --- | --- | --- | --- | --- | --- | --- | --- | --- |
|  | Resistance |  | | Association with antibiotic use | |  |  |  |  |  |
|  | no. of tests/strata | | proportion resistant (95% CI) | odds ratio (95% CI) | p-value | |  |  |  |  |
| Amoxicillin/Ampicillin | 2033 / 123 | | 62 (55 - 68) | 1.16 (0.87 - 1.56) | 0.31 | |  |  |  |  |
| Amoxicillin-clavulanic acid | 2033 / 123 | | 39 (34 - 44) | 1.07 (1.02 - 1.12) | 0.009 | |  |  |  |  |
| Ceftriaxone | 946 / 106 | | 19 (14 - 25) | 1.25 (0.77 - 2.01) | 0.36 | |  |  |  |  |
| Cefepime | 944 / 106 | | 9 (6 - 13) | 1.09 (0.91 - 1.30) | 0.34 | |  |  |  |  |
| Piperacillin-tazobactam | 946 / 106 | | 17 (13 - 22) | 1.96 (1.29 - 2.97) | 0.002 | |  |  |  |  |
| Quinolones | 2033 / 123 | | 20 (18 - 23) | 1.54 (1.30 - 1.83) | <0.001 | |  |  |  |  |
| Trimethoprim-sulfamethoxazole | 2032 / 123 | | 32 (27 - 37) | 1.62 (1.21 - 2.17) | 0.001 | |  |  |  |  |
| Gentamicin | 946 / 106 | | 17 (14 - 22) | 1.19 (0.40 - 3.55) | 0.76 | |  |  |  |  |
| Combined | 11913 / 916 | | 24 (16 - 35) | 1.09 (1.03 - 1.14) | 0.002 | |  |  |  |  |
| Results from logistic mixed effects models. The odds ratios indicate the relative change in the odds for resistance for an increase in last year's antibiotic use by 5 DDD/100 bed-days. Strata refers to year/department combinations, CI: confidence interval. | | | | | | |  |  |  |  |

| **Table S3.** Antibiotic resistance and association with antibiotic use for nosocomial and community *E. coli* | | | | | | |  |  |  |  |  |
| --- | --- | --- | --- | --- | --- | --- | --- | --- | --- | --- | --- |
|  | Resistance |  | Association with antibiotic use | |  |  |  |  |  |  |  |
|  | no. of tests/strata | proportion resistant (95% CI) | odds ratio (95% CI) | p-value | p-value for interaction | |  |  |  |  |  |
| Amoxicillin/Ampicillin |  |  |  |  | 0.73 | |  |  |  |  |  |
| community | 1949 / 137 | 51 (46 - 56) | 1.10 (0.84 - 1.43) | 0.50 |  | |  |  |  |  |  |
| nosocomial | 2330 / 139 | 62 (55 - 69) | 1.08 (0.84 - 1.40) | 0.53 |  | |  |  |  |  |  |
| Amoxicillin-clavulanic acid |  |  |  |  | 0.11 | |  |  |  |  |  |
| community | 1949 / 137 | 29 (25 - 33) | 1.01 (0.97 - 1.07) | 0.58 |  | |  |  |  |  |  |
| nosocomial | 2330 / 139 | 39 (35 - 44) | 1.07 (1.02 - 1.12) | 0.004 |  | |  |  |  |  |  |
| Ceftriaxone |  |  |  |  | 0.20 | |  |  |  |  |  |
| community | 842 / 108 | 13 (8 - 21) | 1.96 (0.93 - 4.14) | 0.08 |  | |  |  |  |  |  |
| nosocomial | 1085 / 119 | 18 (13 - 24) | 1.10 (0.68 - 1.76) | 0.71 |  | |  |  |  |  |  |
| Cefepime |  |  |  |  | 0.52 | |  |  |  |  |  |
| community | 837 / 107 | 6 (3 - 12) | 1.23 (0.91 - 1.66) | 0.19 |  | |  |  |  |  |  |
| nosocomial | 1083 / 119 | 8 (6 - 12) | 1.06 (0.90 - 1.25) | 0.49 |  | |  |  |  |  |  |
| Piperacillin-tazobactam |  |  |  |  | 0.034 | |  |  |  |  |  |
| community | 841 / 108 | 12 (9 - 16) | 1.10 (0.67 - 1.80) | 0.70 |  | |  |  |  |  |  |
| nosocomial | 1085 / 119 | 17 (13 - 21) | 2.11 (1.45 - 3.07) | <0.001 |  | |  |  |  |  |  |
| Quinolones |  |  |  |  | 0.12 | |  |  |  |  |  |
| community | 1949 / 137 | 17 (13 - 21) | 1.12 (0.83 - 1.49) | 0.46 |  | |  |  |  |  |  |
| nosocomial | 2330 / 139 | 20 (17 - 23) | 1.52 (1.25 - 1.86) | <0.001 |  | |  |  |  |  |  |
| Trimethoprim-sulfamethoxazole |  |  |  |  | 0.15 | |  |  |  |  |  |
| community | 1948 / 137 | 25 (23 - 29) | 1.32 (1.08 - 1.61) | 0.007 |  | |  |  |  |  |  |
| nosocomial | 2329 / 139 | 32 (27 - 37) | 1.59 (1.19 - 2.13) | 0.002 |  | |  |  |  |  |  |
| Gentamicin |  |  |  |  | 0.55 | |  |  |  |  |  |
| community | 841 / 108 | 18 (12 - 24) | 0.67 (0.13 - 3.48) | 0.64 |  | |  |  |  |  |  |
| nosocomial | 1085 / 119 | 18 (14 - 22) | 1.30 (0.45 - 3.71) | 0.63 |  | |  |  |  |  |  |
| Combined |  |  |  |  | 0.07 | |  |  |  |  |  |
| community | 11156 / 979 | 19 (13 - 29) | 1.03 (0.98 - 1.09) | 0.27 |  | |  |  |  |  |  |
| nosocomial | 13657 / 1032 | 24 (15 - 36) | 1.09 (1.04 - 1.14) | <0.001 |  | |  |  |  |  |  |
| Results from separate logistic mixed effects models for nosocomial and community *E. coli* (the latter were not part of the main analysis). The odds ratios indicate the relative change in the odds for resistance for an increase in antibiotic use by 5 DDD/100 bed-days. The p-value for interaction is derived from a model with both, nosocomial and community samples. Strata refers to year/department combinations, CI: confidence interval. | | | | | | |  |  |  |  |  |

| **Table S4.** Antibiotic resistance and association with antibiotic use for *E. coli* with an alternative definition of nosocomial. | | | | | | |  |  |  |  |
| --- | --- | --- | --- | --- | --- | --- | --- | --- | --- | --- |
|  | Resistance |  | | Association with antibiotic use | |  |  |  |  |  |
|  | no. of tests/strata | | proportion resistant (95% CI) | odds ratio (95% CI) | p-value | |  |  |  |  |
| Amoxicillin/Ampicillin | 1805 / 133 | | 64 (57 - 70) | 1.15 (0.87 - 1.53) | 0.32 | |  |  |  |  |
| Amoxicillin-clavulanic acid | 1805 / 133 | | 40 (36 - 45) | 1.07 (1.02 - 1.12) | 0.004 | |  |  |  |  |
| Ceftriaxone | 882 / 112 | | 18 (13 - 25) | 1.20 (0.73 - 1.96) | 0.48 | |  |  |  |  |
| Cefepime | 882 / 112 | | 8 (6 - 12) | 1.02 (0.87 - 1.21) | 0.79 | |  |  |  |  |
| Piperacillin-tazobactam | 882 / 112 | | 17 (14 - 21) | 2.06 (1.46 - 2.90) | <0.001 | |  |  |  |  |
| Quinolones | 1805 / 133 | | 20 (18 - 23) | 1.60 (1.35 - 1.90) | <0.001 | |  |  |  |  |
| Trimethoprim-sulfamethoxazole | 1804 / 133 | | 32 (27 - 37) | 1.66 (1.25 - 2.20) | <0.001 | |  |  |  |  |
| Gentamicin | 882 / 112 | | 19 (15 - 24) | 1.22 (0.39 - 3.88) | 0.73 | |  |  |  |  |
| Combined | 10747 / 980 | | 25 (16 - 37) | 1.10 (1.04 - 1.15) | <0.001 | |  |  |  |  |
| Results from logistic mixed effects models for probably nosocomial *E. coli*, isolated more than 5 days after admission. The odds ratio indicates the relative change in the odds for resistance for an increase in antibiotic use by 5 DDD/100 bed-days. Strata refers to year/department combinations, CI: confidence interval. | | | | | | |  |  |  |  |

| **Table S5.** Antibiotic resistance in *E. coli* and association with antibiotic use according to the origin of the sample. | | | | | | |  | | |  |  | |  |  |  |  |  |
| --- | --- | --- | --- | --- | --- | --- | --- | --- | --- | --- | --- | --- | --- | --- | --- | --- | --- |
|  | Resistance |  | Association with antibiotic use | |  |  |  |  |  |  |  |  |  |  |  |  |  |
|  | no. of tests/strata | proportion resistant (95% CI) | odds ratio (95% CI) | p-value | p-value for interaction | |  |  |  |  |  |  |  |  |  |  |  |
| Amoxicillin/Ampicillin |  |  |  |  | 0.68 | |  |  |  |  |  |  |  |  |  |  |  |
| urine | 1503 / 131 | 60 (52 - 68) | 1.01 (0.76 - 1.36) | 0.93 |  | |  |  |  |  |  |  |  |  |  |  |  |
| blood | 264 / 71 | 68 (61 - 74) | 1.04 (0.60 - 1.80) | 0.89 |  | |  |  |  |  |  |  |  |  |  |  |  |
| other | 563 / 93 | 69 (62 - 76) | 1.20 (0.81 - 1.78) | 0.37 |  | |  |  |  |  |  |  |  |  |  |  |  |
| Amoxicillin-clavulanic acid |  |  |  |  | 0.31 | |  |  |  |  |  |  |  |  |  |  |  |
| urine | 1503 / 131 | 37 (33 - 42) | 1.10 (1.05 - 1.16) | <0.001 |  | |  |  |  |  |  |  |  |  |  |  |  |
| blood | 264 / 71 | 42 (34 - 50) | 1.05 (0.97 - 1.14) | 0.24 |  | |  |  |  |  |  |  |  |  |  |  |  |
| other | 563 / 93 | 44 (35 - 54) | 1.02 (0.94 - 1.11) | 0.62 |  | |  |  |  |  |  |  |  |  |  |  |  |
| Ceftriaxone |  |  |  |  | 0.45 | |  |  |  |  |  |  |  |  |  |  |  |
| urine | 258 / 88 | 40 (32 - 48) | 1.24 (0.71 - 2.15) | 0.45 |  | |  |  |  |  |  |  |  |  |  |  |  |
| blood | 264 / 71 | 10 (7 - 15) | 2.24 (1.19 - 4.24) | 0.013 |  | |  |  |  |  |  |  |  |  |  |  |  |
| other | 563 / 93 | 6 (3 - 14) | 0.76 (0.23 - 2.49) | 0.65 |  | |  |  |  |  |  |  |  |  |  |  |  |
| Cefepime |  |  |  |  | 0.73 | |  |  |  |  |  |  |  |  |  |  |  |
| urine | 256 / 88 | 15 (9 - 23) | 1.18 (0.91 - 1.52) | 0.22 |  | |  |  |  |  |  |  |  |  |  |  |  |
| blood | 264 / 71 | 6 (3 - 11) | 1.01 (0.85 - 1.21) | 0.88 |  | |  |  |  |  |  |  |  |  |  |  |  |
| other | 563 / 93 | 3 (1 - 9) | 1.13 (0.77 - 1.67) | 0.53 |  | |  |  |  |  |  |  |  |  |  |  |  |
| Piperacillin-tazobactam |  |  |  |  | 0.08 | |  |  |  |  |  |  |  |  |  |  |  |
| urine | 258 / 88 | 20 (16 - 26) | 1.30 (0.80 - 2.09) | 0.29 |  | |  |  |  |  |  |  |  |  |  |  |  |
| blood | 264 / 71 | 12 (7 - 20) | 1.51 (0.76 - 3.02) | 0.24 |  | |  |  |  |  |  |  |  |  |  |  |  |
| other | 563 / 93 | 15 (12 - 19) | 2.46 (1.94 - 3.12) | <0.001 |  | |  |  |  |  |  |  |  |  |  |  |  |
| Quinolones |  |  |  |  | 0.86 | |  |  |  |  |  |  |  |  |  |  |  |
| urine | 1503 / 131 | 19 (16 - 22) | 1.58 (1.32 - 1.89) | <0.001 |  | |  |  |  |  |  |  |  |  |  |  |  |
| blood | 264 / 71 | 25 (17 - 34) | 1.40 (0.87 - 2.26) | 0.17 |  | |  |  |  |  |  |  |  |  |  |  |  |
| other | 563 / 93 | 23 (17 - 30) | 1.65 (1.13 - 2.40) | 0.009 |  | |  |  |  |  |  |  |  |  |  |  |  |
| Trimethoprim-sulfamethoxazole |  |  |  |  | 0.38 | |  |  |  |  |  |  |  |  |  |  |  |
| urine | 1503 / 131 | 31 (28 - 35) | 1.77 (1.41 - 2.23) | <0.001 |  | |  |  |  |  |  |  |  |  |  |  |  |
| blood | 264 / 71 | 38 (27 - 51) | 1.95 (1.18 - 3.23) | 0.009 |  | |  |  |  |  |  |  |  |  |  |  |  |
| other | 562 / 93 | 33 (27 - 40) | 1.35 (0.89 - 2.04) | 0.16 |  | |  |  |  |  |  |  |  |  |  |  |  |
| Gentamicin |  |  |  |  | 0.030 | |  |  |  |  |  |  |  |  |  |  |  |
| urine | 258 / 88 | 37 (29 - 45) | 0.72 (0.12 - 4.29) | 0.71 |  | |  |  |  |  |  |  |  |  |  |  |  |
| blood | 264 / 71 | 10 (6 - 14) | 0.01 (0.00 - 1.79) | 0.08 |  | |  |  |  |  |  |  |  |  |  |  |  |
| other | 563 / 93 | 11 (8 - 16) | 2.79 (0.71 - 11.04) | 0.14 |  | |  |  |  |  |  |  |  |  |  |  |  |
| Combined |  |  |  |  | 0.74 | |  |  |  |  |  |  |  |  |  |  |  |
| urine | 7042 / 796 | 31 (22 - 42) | 1.12 (1.06 - 1.19) | <0.001 |  | |  |  |  |  |  |  |  |  |  |  |  |
| blood | 2112 / 279 | 21 (11 - 37) | 1.07 (0.99 - 1.17) | 0.10 |  | |  |  |  |  |  |  |  |  |  |  |  |
| other | 4503 / 644 | 21 (11 - 37) | 1.08 (1.00 - 1.16) | 0.05 |  | |  |  |  |  |  |  |  |  |  |  |  |
| Results from separate logistic mixed effects models for *E. coli* samples form urine, blood or other tissues. The odds ratios indicate the relative change in the odds for resistance for an increase in antibiotic use by 5 DDD/100 bed-days. The p-value for interaction is derived from a model with all origins. Strata refers to year/department combinations, CI: confidence interval. | | | | | | | |  |  | | |  | | | |  |  |

| **Table S6.** Antibiotic resistance and association with last year's antibiotic use for *K. pneumoniae* | | | | | | |  | |  | |  | | |  |
| --- | --- | --- | --- | --- | --- | --- | --- | --- | --- | --- | --- | --- | --- | --- |
|  | Resistance | |  | Association with antibiotic use | |  |  |  |  |  |  |  |  |  |
|  | no. of tests/strata | proportion resistant (95% CI) | | odds ratio (95% CI) | p-value | |  |  |  |  |  |  |  |  |
| Amoxicillin-clavulanic acid | 555 / 100 | 17 (14 - 22) | | 1.05 (0.98 - 1.14) | 0.16 | |  |  |  |  |  |  |  |  |
| Ceftriaxone | 319 / 79 | 13 (7 - 21) | | 1.38 (0.62 - 3.07) | 0.43 | |  |  |  |  |  |  |  |  |
| Cefepime | 319 / 79 | 3 (1 - 10) | | 0.87 (0.61 - 1.24) | 0.44 | |  |  |  |  |  |  |  |  |
| Piperacillin-tazobactam | 319 / 79 | 21 (17 - 26) | | 0.86 (0.60 - 1.22) | 0.39 | |  |  |  |  |  |  |  |  |
| Quinolones | 555 / 100 | 6 (4 - 10) | | 1.47 (0.99 - 2.18) | 0.06 | |  |  |  |  |  |  |  |  |
| Trimethoprim-sulfamethoxazole | 555 / 100 | 17 (13 - 21) | | 2.17 (1.50 - 3.12) | <0.001 | |  |  |  |  |  |  |  |  |
| Gentamicin | 319 / 79 | 9 (4 - 20) | | 0.68 (0.06 - 7.98) | 0.76 | |  |  |  |  |  |  |  |  |
| Combined | 2941 / 616 | 12 (9 - 17) | | 1.05 (0.97 - 1.13) | 0.27 | |  |  |  |  |  |  |  |  |
| Results from logistic mixed effects models. The odds ratios represent the change in the odds for resistance for an increase in last year's antibiotic use by 5 DDD/100 bed-days. Strata refers to year/department combinations, CI: confidence interval. | | | | | | |  |  | |  | |  |  |  |

| **Table S7.** Antibiotic resistance and association with antibiotic use for nosocomial and community *K. pneumoniae* | | | | | | |  |  |  |  |  |
| --- | --- | --- | --- | --- | --- | --- | --- | --- | --- | --- | --- |
|  | Resistance |  | Association with antibiotic use | |  |  |  |  |  |  |  |
|  | no. of tests/strata | proportion resistant (95% CI) | odds ratio (95% CI) | p-value | p-value for interaction | |  |  |  |  |  |
| Amoxicillin-clavulanic acid |  |  |  |  | 0.44 | |  |  |  |  |  |
| community | 362 / 106 | 14 (11 - 18) | 1.04 (0.97 - 1.10) | 0.25 |  | |  |  |  |  |  |
| nosocomial | 648 / 115 | 17 (14 - 21) | 1.07 (1.01 - 1.14) | 0.025 |  | |  |  |  |  |  |
| Ceftriaxone |  |  |  |  | 0.75 | |  |  |  |  |  |
| community | 147 / 75 | 13 (5 - 27) | 1.32 (0.50 - 3.43) | 0.58 |  | |  |  |  |  |  |
| nosocomial | 369 / 92 | 10 (5 - 18) | 1.12 (0.52 - 2.45) | 0.77 |  | |  |  |  |  |  |
| Cefepime |  |  |  |  | 0.26 | |  |  |  |  |  |
| community | 147 / 75 | 9 (5 - 15) | 1.18 (0.88 - 1.56) | 0.27 |  | |  |  |  |  |  |
| nosocomial | 369 / 92 | 3 (1 - 9) | 0.86 (0.59 - 1.25) | 0.43 |  | |  |  |  |  |  |
| Piperacillin-tazobactam |  |  |  |  | 0.51 | |  |  |  |  |  |
| community | 147 / 75 | 17 (9 - 29) | 0.58 (0.25 - 1.36) | 0.21 |  | |  |  |  |  |  |
| nosocomial | 369 / 92 | 21 (17 - 25) | 0.84 (0.61 - 1.18) | 0.32 |  | |  |  |  |  |  |
| Quinolones |  |  |  |  | 0.80 | |  |  |  |  |  |
| community | 362 / 106 | 8 (5 - 11) | 1.42 (1.04 - 1.96) | 0.029 |  | |  |  |  |  |  |
| nosocomial | 648 / 115 | 6 (3 - 9) | 1.37 (0.94 - 2.01) | 0.10 |  | |  |  |  |  |  |
| Trimethoprim-sulfamethoxazole |  |  |  |  | 0.37 | |  |  |  |  |  |
| community | 362 / 106 | 13 (9 - 19) | 1.62 (1.08 - 2.42) | 0.021 |  | |  |  |  |  |  |
| nosocomial | 648 / 115 | 15 (12 - 19) | 2.02 (1.44 - 2.84) | <0.001 |  | |  |  |  |  |  |
| Gentamicin |  |  |  |  | 0.77 | |  |  |  |  |  |
| community | 147 / 75 | 11 (4 - 26) | 0.98 (0.05 - 19.20) | 0.99 |  | |  |  |  |  |  |
| nosocomial | 369 / 92 | 8 (3 - 16) | 1.82 (0.13 - 25.35) | 0.66 |  | |  |  |  |  |  |
| Combined |  |  |  |  | 0.58 | |  |  |  |  |  |
| community | 1674 / 618 | 12 (9 - 15) | 1.05 (0.98 - 1.13) | 0.15 |  | |  |  |  |  |  |
| nosocomial | 3420 / 713 | 12 (8 - 16) | 1.06 (0.99 - 1.14) | 0.07 |  | |  |  |  |  |  |
| Results from separate logistic mixed effects models for nosocomial and community *K. pneumoniae* (the latter were not part of the main analysis). The odds ratios indicate the relative change in the odds for resistance for an increase in antibiotic use by 5 DDD/100 bed-days. The p-value for interaction is derived from a model with both, nosocomial and community samples. Strata refers to year/department combinations, CI: confidence interval. | | | | | | |  |  |  |  |  |

| **Table S8.** Antibiotic resistance and association with antibiotic use for *K. pneumoniae* with an alternative definition of nosocomial. | | | | | | |  | |  | |  | | |  |
| --- | --- | --- | --- | --- | --- | --- | --- | --- | --- | --- | --- | --- | --- | --- |
|  | Resistance | |  | Association with antibiotic use | |  |  |  |  |  |  |  |  |  |
|  | no. of tests/strata | proportion resistant (95% CI) | | odds ratio (95% CI) | p-value | |  |  |  |  |  |  |  |  |
| Amoxicillin-clavulanic acid | 524 / 102 | 18 (14 - 21) | | 1.08 (1.02 - 1.15) | 0.009 | |  |  |  |  |  |  |  |  |
| Ceftriaxone | 305 / 84 | 8 (3 - 16) | | 1.19 (0.48 - 2.98) | 0.71 | |  |  |  |  |  |  |  |  |
| Cefepime | 305 / 84 | 1 (0 - 11) | | 0.95 (0.54 - 1.67) | 0.87 | |  |  |  |  |  |  |  |  |
| Piperacillin-tazobactam | 305 / 84 | 20 (16 - 25) | | 0.91 (0.63 - 1.30) | 0.60 | |  |  |  |  |  |  |  |  |
| Quinolones | 524 / 102 | 5 (3 - 9) | | 1.39 (0.90 - 2.14) | 0.14 | |  |  |  |  |  |  |  |  |
| Trimethoprim-sulfamethoxazole | 524 / 102 | 14 (10 - 18) | | 2.27 (1.56 - 3.31) | <0.001 | |  |  |  |  |  |  |  |  |
| Gentamicin | 305 / 84 | 6 (2 - 17) | | 3.68 (0.23 - 58.90) | 0.36 | |  |  |  |  |  |  |  |  |
| Combined | 2792 / 642 | 10 (7 - 15) | | 1.09 (1.02 - 1.18) | 0.012 | |  |  |  |  |  |  |  |  |
| Results from logistic mixed effects models for probably nosocomial *K. pneumoniae*, isolated more than 5 days after admission. The odds ratio indicates the relative change in the odds for resistance for an increase in antibiotic use by 5 DDD/100 bed-days. Strata refers to year/department combinations, CI: confidence interval. | | | | | | |  |  | |  | |  |  |  |

| **Table S9.** Antibiotic resistance in *K. pneumoniae* and association with antibiotic use according to the origin of the sample. | | | | | | |
| --- | --- | --- | --- | --- | --- | --- |
|  | Resistance |  | Association with antibiotic use | |  |  |
|  | no. of tests/strata | proportion resistant (95% CI) | odds ratio (95% CI) | p-value | p-value for interaction | |
| Amoxicillin-clavulanic acid |  |  |  |  | 0.54 | |
| urine | 329 / 97 | 13 (9 - 19) | 1.09 (1.00 - 1.19) | 0.05 |  | |
| blood | 89 / 36 | 22 (14 - 32) | 1.12 (0.93 - 1.36) | 0.22 |  | |
| other | 230 / 75 | 21 (16 - 27) | 1.02 (0.90 - 1.15) | 0.76 |  | |
| Ceftriaxone |  |  |  |  | 0.26 | |
| urine | 51 / 40 | 38 (21 - 59) | 3.03 (0.62 - 14.74) | 0.17 |  | |
| blood | 89 / 36 | 4 (1 - 14) | 0.38 (0.04 - 3.87) | 0.41 |  | |
| other | 229 / 74 | 7 (4 - 11) | 1.41 (0.73 - 2.74) | 0.31 |  | |
| Cefepime |  |  |  |  | 0.76 | |
| urine | 51 / 40 | 17 (9 - 30) | 0.87 (0.48 - 1.59) | 0.65 |  | |
| blood | 89 / 36 | 0 (0 - 76) | 1.19 (0.55 - 2.60) | 0.65 |  | |
| other | 229 / 74 | 1 (0 - 19) | 0.83 (0.46 - 1.48) | 0.52 |  | |
| Piperacillin-tazobactam |  |  |  |  | 0.86 | |
| urine | 51 / 40 | 39 (27 - 53) | 0.75 (0.28 - 1.99) | 0.56 |  | |
| blood | 89 / 36 | 16 (10 - 25) | 1.06 (0.47 - 2.42) | 0.88 |  | |
| other | 229 / 74 | 18 (13 - 24) | 0.95 (0.62 - 1.45) | 0.80 |  | |
| Quinolones |  |  |  |  | 0.68 | |
| urine | 329 / 97 | 7 (4 - 10) | 1.36 (0.93 - 1.99) | 0.11 |  | |
| blood | 89 / 36 | 6 (2 - 22) | 2.26 (0.63 - 8.09) | 0.21 |  | |
| other | 230 / 75 | 6 (2 - 16) | 1.18 (0.58 - 2.39) | 0.65 |  | |
| Trimethoprim-sulfamethoxazole |  |  |  |  | 0.10 | |
| urine | 329 / 97 | 15 (11 - 20) | 1.78 (1.21 - 2.61) | 0.003 |  | |
| blood | 89 / 36 | 18 (10 - 31) | 4.51 (1.79 - 11.40) | 0.001 |  | |
| other | 230 / 75 | 14 (9 - 20) | 1.45 (0.68 - 3.10) | 0.34 |  | |
| Gentamicin |  |  |  |  | 0.62 | |
| urine | 51 / 40 | 33 (21 - 47) | 13.83 (0.20 - 960.71) | 0.22 |  | |
| blood | 89 / 36 | 0 (0 - 100) | 0.40 (0.00 - 3.61e+11) | 0.95 |  | |
| other | 229 / 74 | 8 (5 - 12) | 1.84 (0.39 - 8.71) | 0.44 |  | |
| Combined |  |  |  |  | 0.10 | |
| urine | 1191 / 358 | 21 (13 - 34) | 1.10 (1.03 - 1.18) | 0.006 |  | |
| blood | 623 / 139 | 8 (4 - 17) | 1.14 (0.97 - 1.34) | 0.11 |  | |
| other | 1606 / 487 | 10 (6 - 15) | 1.03 (0.94 - 1.14) | 0.51 |  | |
| Results from separate logistic mixed effects models for *K. pneumoniae* samples form urine, blood or other tissues. The odds ratios indicate the relative change in the odds for resistance for an increase in antibiotic use by 5 DDD/100 bed-days. The p-value for interaction is derived from a model with all origins. Strata refers to year/department combinations, CI: confidence interval. | | | | | | |
